# Interactions between toll‐like receptors signaling pathway and gut microbiota in host homeostasis

**DOI:** 10.1002/iid3.1356

**Published:** 2024-07-29

**Authors:** Luping Chen, Linfang Zhang, Hua Hua, Li Liu, Yuejian Mao, Ruirui Wang

**Affiliations:** ^1^ Shanghai Innovation Center of TCM Health Service Shanghai University of Traditional Chinese Medicine Shanghai China; ^2^ Department of Pharmacology and Toxicology, School of Nutrition and Translational Research in Metabolism Maastricht University Maastricht The Netherlands; ^3^ Oxford Suzhou Centre for Advanced Research Suzhou China; ^4^ Sichuan Institute for Translational Chinese Medicine Chengdu China; ^5^ Sichuan Academy of Chinese Medical Sciences Chengdu China; ^6^ Global R&D Innovation Center Inner Mongolia Mengniu Dairy (Group) Co. Ltd. Hohhot Inner Mongolia China

**Keywords:** colorectal cancer, gut microbiota, inflammatory bowel disease, obesity, TLRs

## Abstract

**Background:**

Toll‐like receptors (TLRs) are a family of fundamental pattern recognition receptors in the innate immune system, constituting the first line of defense against endogenous and exogenous antigens. The gut microbiota, a collection of commensal microorganisms in the intestine, is a major source of exogenous antigens. The components and metabolites of the gut microbiota interact with specific TLRs to contribute to whole‐body immune and metabolic homeostasis.

**Objective:**

This review aims to summarize the interaction between the gut microbiota and TLR signaling pathways and to enumerate the role of microbiota dysbiosis‐induced TLR signaling pathways in obesity, inflammatory bowel disease (IBD), and colorectal cancer (CRC).

**Results:**

Through the recognition of TLRs, the microbiota facilitates the development of both the innate and adaptive immune systems, while the immune system monitors dynamic changes in the commensal bacteria to maintain the balance of the host‐microorganism symbiosis. Dysbiosis of the gut microbiota can induce a cascade of inflammatory and metabolic responses mediated by TLR signaling pathways, potentially resulting in various metabolic and inflammatory diseases.

**Conclusion:**

Understanding the crosstalk between TLRs and the gut microbiota contributes to potential therapeutic applications in related diseases, offering new avenues for treatment strategies in conditions like obesity, IBD, and CRC.

## Graphical abstract

Recent studies have revealed that gut microbiota plays a vital role in host homeostasis and shows a close relationship with various metabolic diseases. The interactions between toll‐like receptors (TLRs) and gut microbiota contribute to whole‐body homeostasis in a complex manner. Dysbiosis of gut microbiota could induce a cascade of inflammatory responses mediated by TLR signaling pathways, which may result in metabolic and inflammatory diseases. We summarize the interaction between gut microbiota and TLR signaling pathways and then enumerate the role of microbiota dysbiosis‐induced TLR signaling pathways in obesity and inflammatory bowel disease to better understand how the crosstalk between TLRs and gut microbiota contributes to potential therapeutic applications in related diseases.

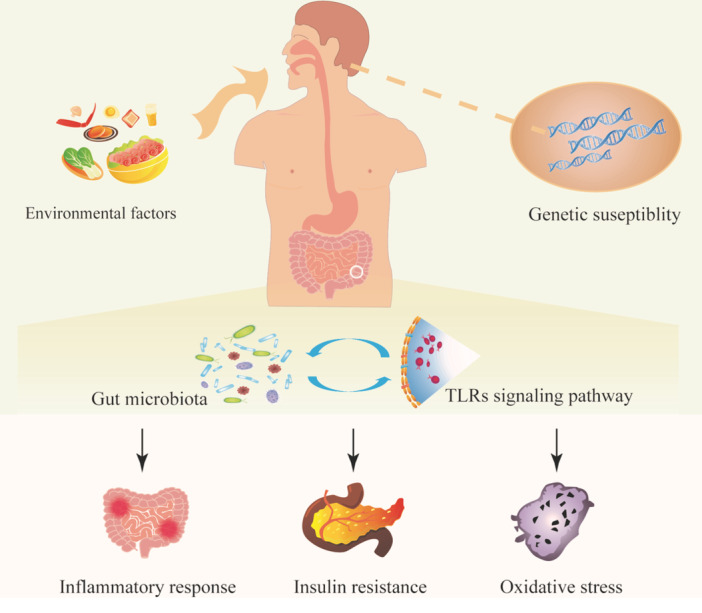

## 1 INTRODUCTION

Toll‐like receptors (TLRs) are pattern recognition receptors (PRRs) capable of being activated by a wide array of exogenous and endogenous pathogenic molecules, considered vital constituents of the innate immune system.^1^ TLRs are widely distributed on immune cells such as macrophages, dendritic cells (DCs), natural killer cells, mast cells, as well as nonimmune cells like epithelial cells.^2^ Currently, there are 10 TLR subtypes (TLR1–TLR10) identified in humans and 12 TLR subtypes (TLR1–TLR9, TLR11–TLR13) identified in mice.^3^ TLR1, TLR2, TLR4, TLR5, and TLR6 are expressed on the cell membrane, while TLR3, TLR7, and TLR9 are primarily expressed on endosomes.^4^ As important sensors for pathogen‐associated molecular patterns (PAMPs), all TLRs are composed of similar domains: an ectodomain with leucine‐rich repeats for PAMP recognition, a transmembrane domain, and a cytoplasmic toll/IL‐1 receptor domain responsible for initiating downstream signaling pathways.^5^

The precise immune response mediated by TLRs is crucial for organismal survival amidst significant infection risks.^6^ However, the dramatic changes in modern environments and lifestyles have led to an increase in chronic diseases rather than infectious ones. It is recognized that systemic, persistent, low‐grade inflammation is a central feature of various metabolic and inflammatory diseases, commonly referred to as chronic inflammation. The specific triggers initiating the cascade of chronic inflammation mediated by TLRs remain elusive.

The gut microbiota is considered as an external “metabolic and immune organ,” playing a pivotal role not only in nutrient and energy absorption from food but also as a significant source of antigens that interact with their corresponding receptors to regulate the host immune system and activate adaptive immune cells.^7^ Recent research has illustrated that there is a bidirectional and dynamic balance between gut microbiota and host immune system, crucial for maintaining host health. The host immune system influences the composition of the gut microbiota, while the gut microbiota aids in immune system maturation and regulation of immune responses.^8^ Studies with germ‐free mice have revealed that early exposure to microbes is essential for proper immune system development, as germ‐free mice exhibit immature immune organs and aberrant immune signaling.^9^, ^10^, ^11^, ^12^ The interface between gut microbiota and TLRs can be considered as a core homeostatic mechanism, the dysfunction of which could lead to various chronic metabolic disorders.

This review emphasizes the interaction between gut microbiota and TLRs signaling pathways. Additionally, it delineates the role of microbiota dysbiosis‐induced TLRs signaling in chronic metabolic disorders, including obesity and inflammatory bowel disease (IBD), and further discusses potential gut microbiota‐targeted therapeutics.

## 2 TLRS AND GUT MICROBIOTA: INTERACTIONS AND CROSSTALKS

**Figure 1:**
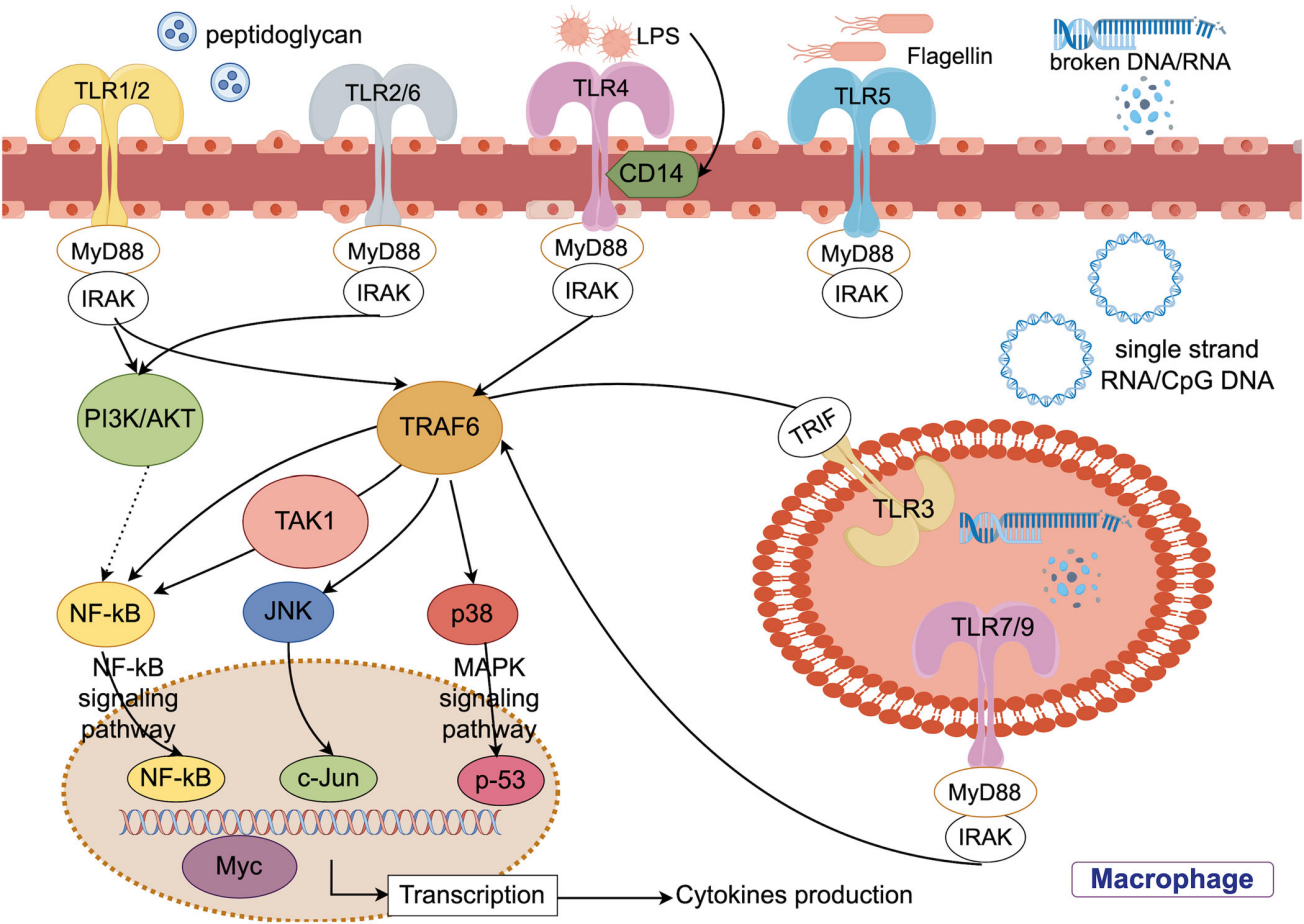
Toll‐like receptors (TLRs) in gut dysbiosis/health. Interaction between microbiota and TLRs shows that gram‐positive bacteria‐derived lipoproteins and peptidoglycan stimulate TLR2, gram‐negative bacteria‐derived lipopolysaccharides (LPS) spur TLR4, whip‐like flagellin respond to TLR5, single‐stranded RNA, along with damaged DNA, stimulate TLR7 and TLR9. Once TLRs combine with their respective ligands, the downstream signaling molecules will be recruited through the myeloid differentiation factor (MyD88) pathway or MyD88‐independent signaling transduction to activate multiple pathways, including classical NF‐κB and MAPKs signaling pathways, promoting to encode tumorigenic proteins or inflammatory cytokines. Figure 1 is created by Figdraw.

PRRs known as TLRs function as the sentinels of the innate immune system, detecting microbial‐related antigens within the intestine. Over the course of long‐term evolution, intricate mechanisms have established a delicate equilibrium between the immune system and gut microbiota. Bacterial debris or products have the capacity to engage specific TLRs, initiating downstream reactions. Upon binding with their specific ligands, TLRs initiate downstream signaling cascades by recruiting signaling molecules through the myeloid differentiation factor (MyD88) pathway or MyD88‐independent signaling transduction.^13^ This activation leads to the stimulation of various pathways, such as the classical NF‐κB and mitogen‐activated protein kinases signaling pathways, ultimately resulting in the upregulation of inflammatory cytokine expression (Figure 1). The symbiotic relationship between the TLR signaling pathway and gut microbiota is essential for maintaining host homeostasis. Table 1 lists the TLR signaling pathway and bacteria‐derived ligands.

**Table 1:** Toll‐like receptor (TLR) signaling pathway and bacteria‐derived ligands.

TLRs	Ligands (exogenous/endogenous)	Ligands name	Pathway	Reference
TLR1/2	Exogenous	Pam(3)Cys‐Ser‐(Lys)(4) trihydrochloride (Pam3CKS4)	NF‐κB and MAPK signaling pathway	[14, 15]
TLR2/6	Exogenous	Macrophage‐activating lipopeptide‐2 (MALP‐2)	NF‐kB signaling pathway	[16]
TLR3	Endogenous	Polyinosinic: polycytidylic acid (polyI:C)	TLR3‐TRIF signaling pathway/NF‐kB signaling pathway	[17, 18, 19]
TLR4	Exogenous	lipopolysaccharide (LPS)	TLR‐MyD88 mediated pathways	[20, 21]
TLR5	Exogenous	flagellin	TLR5‐MyD88/NF‐kB signaling pathway	[22, 23]
TLR7/9	Endogenous	Imiquimod (R837)/Resiquimod (R848)/miR‐1983/CpG oligonucleotide (ODN)	NF‐κB/MAPK signaling pathway	[24, 25, 26]

### 2.1 Interaction between TLR2 and gut microbiota

TLR2 expression is responsible for recognizing various microbial components, including lipoproteins derived from Gram‐positive bacteria, peptidoglycan, and lipoteichoic acid, which can promote inflammatory responses and metabolic adaptation.^27^, ^28^ To fulfill this function, TLR2 must bind with TLR1 and TLR6, forming a heterodimeric receptor complex expressed on the cell surface.^29^, ^30^ Previous research has demonstrated that TLR2−/− mice under a chow diet condition exhibit a gut microbiota profile characterized by a decrease in *Proteobacteria* and *Bacteroidetes* at the phylum level while showing an increase in *Firmicutes*. At the genus level, there is an increase in *Oscillospira* and *Ruminococcus*.^31^ Additionally, the composition of the intestinal flora can influence TLR2 expression; for example, a combination of *Lactobacillus acidophilus, Bifidobacteria infantis,* and *Bifidobacteria infantum* can enhance TLR2 expression and improve intestinal barrier integrity.^32^

Growing evidence has been reported suggesting that gut microbiota can have a positive effect on gut homeostasis through the TLR2/IL‐10 pathway. TLR2 signaling pathways can induce the expression of IL‐10, a potent immunosuppressive cytokine capable of inhibiting imiquimod‐induced psoriatic skin inflammation.^33^ *Bacteroides fragilis* has been shown to activate TLR2/IL‐10 signaling and reduce levels of tumor necrosis factor alpha (TNF‐α) and IL‐1β, thereby ameliorating dextran sodium sulfate (DSS) induced colitis.^34^

### 2.2 Interaction between TLR4 and gut microbiota

Within the TLR family, TLR4 stands out as the most pivotal member, playing a crucial role in mediating inflammatory responses triggered by lipopolysaccharides (LPS) derived from gram‐negative bacteria. Upon binding with myeloid differentiation 2 (MD2), the dimeric complex TLR4/MD2 is capable of recognizing LPS, facilitated by the delivery of LPS binding protein (LBP) and CD14.^35^ Injection of LPS profoundly boosts the expression of TLR4.^36^ Additionally, studies involving TLR4−/− and CD14 knockout mice have revealed gut dysbiosis characterized by a higher proportion of *Firmicutes* and a lower proportion of *Bacteroidetes*.^37^ This phenomenon may be attributed to the stimulatory effect of TLR4 signaling, which can lead to the upregulation of antimicrobial peptides and thereby exert a direct antibiotic action.^38^

Accumulating evidence suggests that the interaction between TLR4 and gut microbiota serves as the forefront link connecting innate immune responses to chronic inflammation in related diseases. Studies have reported an increase in TLR4 expression in animals with acute colitis as well as chronic conditions such as diabetes and nonalcoholic steatohepatitis^39^, ^40^, ^41^ Notably, knockout or downregulation of TLR4 has been shown to protect animals from obesity‐induced insulin resistance. Additionally, TLR4 expression has been found to be positively correlated with LPS‐producing bacteria and negatively correlated with prebiotics in numerous metabolic disease models.^42^, ^43^ These results suggest that TLR4 may play a stimulatory role in regulating metabolic diseases through its mediation of disturbed gut microbiota.

### 2.3 Interaction between TLR5 and gut microbiota

TLR5 is stimulated by the bacteria flagellin. It has been illustrated that neonatal TLR5 expression strongly influences the composition of gut microbiota throughout life; thus, the adult beneficial microbiota are shaped during early infancy.^44^ The loss of TLR5 in intestinal epithelial cells (IEC) led to low‐grade inflammation, metabolic syndrome, and were prone to develop, and increased susceptibility to colitis development, with alterations observed in localization and levels of fecal LPS and flagellin in mice.^45^, ^46^, ^47^ Research has indicated that TLR5 is capable of recognizing L‐form bacteria containing flagellin from *Pseudomonas aeruginosa* and *Bacillus subtili*.^48^ Aside from this, *Salmonella Typhimurium* flagellin activates TLR5 to induce cytokine production and chemokine release.^49^ Following the recognition of flagellin by TLR5, immunity is triggered to clear the pathogen.^50^ Conversely, the transmission of TLR signals will be impeded if the immune response to *E. coli* flagellin is hindered by TLR5 deficiency.^51^ In some cases, bacteria evade TLR5 sensing and recognition by downregulating flagellin expression or mutating flagellin molecules.^52^ Recently, flagellin has been developed as an adjuvant to vaccines, and a molecular approach to docking vaccines to TLR5 was used to determine the best vaccine pose.^53^, ^54^ In summary, TLR5 plays a nonredundant role in generating antiflagellin antibody responses that regulate intestinal microbial composition and motility.

### 2.4 Interaction between TLR7 and gut microbiota

TLR7 could recognize resident microbiota and promote protective immunity. Resiquimod (R848), a synthetic TLR7 agonist, can trigger IL‐23 and IL‐22 production, causing *Reg*3*g* expression and restoration of colonization resistance against vancomycin‐resistant *Enterococcus*.^55^ Moreover, TLR7 mediated interferon‐β production proves beneficial in ameliorating gut inflammation by antagonizing TLR4‐related TNF‐α and IL‐6 secretion.^56^ Conversely, some argue that TLR7 is not good for gut homeostasis. Transgenic overexpression of TLR7 is linked to *Lactobacilli* translocation, specifically *L. reuteri* to the liver or mesenteric lymph nodes in wild‐type mice poststimulation with the TLR7 agonist imiquimod. Wild‐type, but not TLR7 KO, C57BL/6 mice exhibit increased gut leakiness when exposed to microbiota from TLR7 transgenic mice.^57^ Thus, TLR7 exhibits dual and controversial effects on maintaining host homeostasis.

### 2.5 Interaction between TLR9 and gut microbiota

TLR9 recognizes PAMPs‐associated unmethylated CpG DNA. Studies indicate that TLR9 agonists elicit a robust type I interferon response in the sigmoid colon, affecting the alpha diversity of gut microbiota but without altering the overall microbial community structure.^58^ However, conflicting findings suggest that commensal *Lactobacillus* can activate TLR9, recruiting classical DCs and releasing IL‐10 and TGF‐β.^59^ Activation of apical TLR9 on IECs by microbiota‐derived signals increases the IFN‐γ/IL‐13 and IL‐10/IL‐13 ratio while suppressing pro‐inflammatory cytokine production (IL‐6, IL‐8, and TNF‐α), thereby playing a crucial role in preventing allergic inflammation.^60^ *Bacteroides thetaiotaomicron* and *Lactobacillus johnsonii* enhance TLR9 expression and activate chitinase‐like protein‐1, promoting the degradation of Candida species' cell walls and attenuating DSS‐induced colitis.^61^ Overall, TLR9 primarily functions in anti‐inflammation and immunosuppression.

## 3 DISEASES MEDIATED BY GUT MICROBIOTA–TLRS INTERPLAY

**Figure 2:**
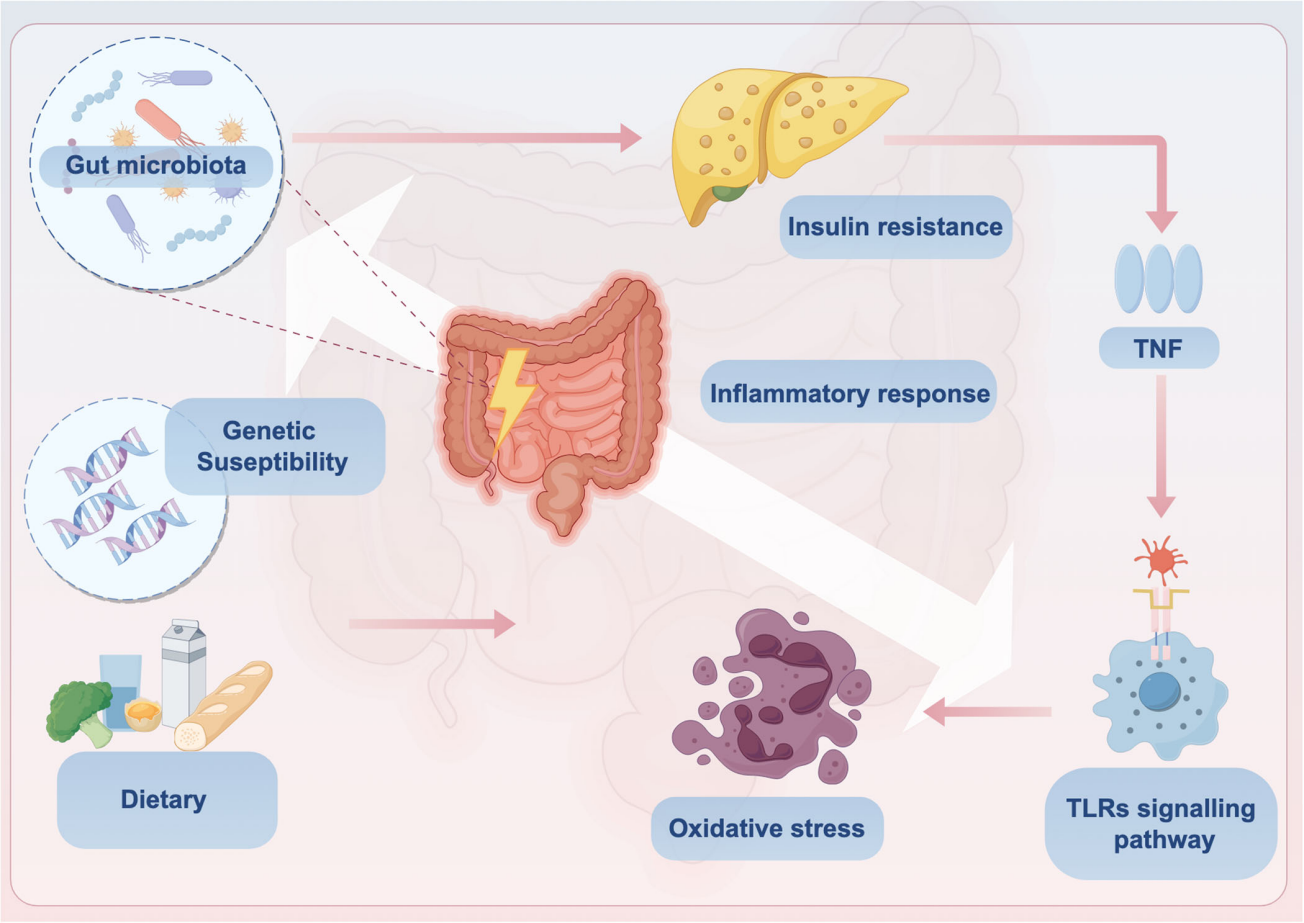
The balance between host health and diseases influenced by gut microbiota and toll‐like receptors (TLRs). Normal interactions between TLRs signaling pathway and gut microbiota play a vital role in host homeostasis, which was affected by environmental factors and genetic susceptibility. When the stable and tight interplays were excessively stimulated beyond host self‐regulation, health balance would be broken and pathogenesis would happen. Overact immune response could trigger inflammatory response, insulin resistance, and oxidative stress. As a result, relative diseases will defeat host health and show up subsequently. TNF, tumor necrosis factor. Figure 2 is created by Figdraw.

TLRs and microbiota have been proven to have a fundamental effect on host health. In this regard, once these stable and tight interactions are excessively stimulated beyond host self‐regulation, the balance of health would be disrupted, leading to pathogenesis (refer to Figure 2). Overexpression of cytokines in response to other functional cells can lead to cellular metabolic malfunction or stimulate relative immune cells, promoting their proliferation and differentiation. Sometimes, excessive microbiota–TLRs signaling results in diseases such as obesity, IBD, and colorectal cancer (CRC). Table 2 summarizes the alternations of gut microbiota, TLR signaling pathways, and related disease.

**Table 2:** The alternations of gut microbiota, toll‐like receptor (TLR) signaling pathways, and related disease.

Gut microbiota	Changes (increase/decrease)	TLR signaling pathway	Changes (activate/inhibit)	Disease	Reference
*Barnesiella, Lactobacillus, Ruminococcus*, and *Flavonifractor*	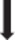	TLR‐4/NF‐κB signaling pathway	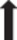	Inflammatory bowel disease	[62]
*Actinobacteria, Clostridium butyricum, Lactobacillus johnsonii, Lactobacillus murinus*, and *Lachnospiraceae bacterium mt14*	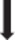	TLR‐4/NF‐κB signaling pathway	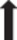	Necrotizing Enterocolitis	[63]
*Alloprevotella*，*bacterium_f_Muribaculaceae*	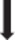	LPS/TLR4/NF‐κB signaling pathway	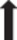	Nonalcoholic fatty liver disease	[64]
*Akkermansia*, *Lactobacillus*, and *A2*.	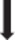	TLR2/NLRP3 signaling pathway	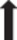	Nonalcoholic Steatohepatitis	[65]
*Alistipes, Lactobacillus*	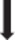	TLR‐MyD88‐NF‐κB signaling pathway	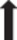	Colorectal Carcinoma	[66]

### 3.1 Gut microbiota–TLRs interaction in obesity

Obese patients have been characterized by excessive fat accumulation, chronic low‐grade inflammation, and insulin resistance. Growing evidence indicates that obesity is accompanied by dysbiosis of gut microbiota, although no consensus has been formed on. It has been reported that a decrease in *Bacteroidetes* and an increase in *Firmicutes* are related to obesity.^67^ A cohort study following weight‐loss bariatric surgery found patients with less favorable outcomes had oral microbiota enriched in phylum *Actinobacteria* and intestinal microbiota enriched in phylum *Bacteroidetes*.^68^ Nirmalkar et al. further showed that the genus *Lactobacillus* and family *Coriobacteriaceae* were enriched in children, and genera *Collinsella* and *Prevotella* were enriched in obese adolescents.^69^ Furthermore, enrichment in the genus *Clostridium* and the species *Eubacterium rectale*, *Clostridium coccoides*, *Lactobacillus reuteri*, *Akkermansia muciniphila*, *Clostridium histolyticum*, and *Staphylococcus aureus* is closely correlated with obese phenotype.^70^ Additionally, TLRs‐induced inflammation plays an important role in obesity, and metabolic syndrome, especially TLR2 and TLR4. The concentration of TLR2 in obese individuals was significantly higher compared to lean individuals.^71^ Furthermore, in obese mice, FoxO1 signaling through TLR4 promotes inflammation in adipose tissue.^72^

Increasing evidence shows that gut microbiota–TLRs interaction contributes to the progression of obesity, especially since activation of TLRs signaling has been recognized as an alternative activator of obesity‐induced inflammation.^73^ However, several studies have investigated the feasibility of using probiotics such as *Akkermansia muciniphila* and *Bifidobacterium spp.* as therapeutics, finding that their administration can ameliorate obesity‐associated metabolic endotoxemia and inflammation.^74^, ^75^ Changes in gut microbiota, such as the increases in *Bacteroidetes, Clostridia, Lactobacillales,* and *Prevotellaceae,* along with reduction in *Bacteroidales, Lachnospiraceae, Rikenellaceae*, and *Desulfovibrio*, can improve systemic inflammation and insulin resistance by reducing plasma LBP and inhibiting TLR4/TRAF6/JUNK signaling.^76^

Oral administration with *Lactobacillus paracasei N1115* or *Akkermansia muciniphila* can inhibit the activation of the LPS/TLR4 signaling pathway and reduce the release of inflammatory factor and insulin resistance,^77^ particularly *Akkermansia muciniphila* or its *Akkermansia muciniphila*‐derived extracellular vesicles. *Akkermansia muciniphila* not only corrects gut permeability and reduces pro‐inflammatory cytokines but also ameliorates defects in learning and memory in high‐fat diet (HFD) induced obese mice. Pharmacologic blockade of TLR4 signaling or antibiotic, treatment, effectively prevents learning and memory deficits in HFD‐fed mice. Thus, gut microbiota plays an unexpected role in cognitive dysfunction in obesity.^78^ These data suggest that the crosstalk between gut microbiota and TLR signaling pathway provides a potential causal link in obesity.

### 3.2 Gut Microbiota–TLRs interaction in IBD

IBD is a chronic condition characterized by idiopathic inflammation and mucosal destruction in the intestine. It consists of two main subtypes, ulcerative colitis (UC) and Crohn's disease (CD). Research indicates that individuals with IBD exhibit significant microbial dysbiosis in the inflamed mucosa.^79^ Notably, tissues from IBD patients have been shown to have an abundance of *Enterobacteriaceae*, *Fusobacteriaceae*, *Pasteurellaceae*, and *Bifidobacteriaceae*.^80^ Eric et al. identified approximately 50 differentially abundant species in IBD. Briefly, *Bifidobacterium breve* and *Clostridium symbiosum* were enriched in UC, while 12 other species were enriched in CD, including *Ruminococcus gnavus*, *Escherichia coli*, and *Clostridium clostridioforme*.^81^ Nevertheless, in UC, *Bifidobacterium breve* and *Clostridium symbiosum* were found to be enriched, compared to non‐IBD controls.^81^ Additionally, studies on colonic tissue from IBD patients have shown enhanced expression of TLR2 and TLR4 but expression of TLR5 was significantly lower, indicating their ability to respond to distinct bacterial products.^82^, ^83^, ^84^ Furthermore, both colon epithelial cells and inflammatory cells in UC patients demonstrate higher expression levels of TLR2, TLR4, and TLR9 than in control groups.^85^ However, findings in mice deficient for TLR2, TLR4, TLR5 or TLR9 indicate that they are protective in IBD models.^80^

As a result of the close relationships between gut microbiota, TLRs, and IBD, many studies have explored interventions aimed at modifying the gut microbiota or targeting TLRs for the treatment of IBD patients. For instance, administration of TLR9 agonist DNA‐based immunomodulatory sequence 0150 has been shown to achieve symptomatic remission, mucosal healing, and histological improvement compared with placebo.^86^ Additionally, a probiotic cocktail has been shown to alleviate clinical symptoms and improve histological scores associated with IBD, which was accompanied by reduced expression of TLR4 and NF‐κB.^87^ Despite the robust correlation observed between IBD and the interaction between gut microbiota and TLRs, the precise mechanistic understanding of how TLRs and gut microbiota contribute to IBD pathogenesis remains elusive.

### 3.3 Gut microbiota–TLRs interaction in CRC

CRC ranks as the third leading cause of cancer‐related mortality worldwide.^88^ Recent data indicate a concerning rise in death rates, potentially linked to inadequate healthcare systems and limited awareness of cancer screening protocols.^89^ The ratio of *Firmicutes*, *Bacteroidetes*, and *Proteobacteria* in CRC patients may change, as well as decreased abundance and diversity of gut microbiota.^90^, ^91^ At the same time, *Fusobacterium nucleatum*, *Streptococcus bovis*, *E. coli,* and enterotoxigenic *Bacteroides fragilis* have also been linked to CRC occurrence.^92^ The role of gut microbiota in CRC in inducing inflammation through TLR is emerging, such as IL‐1β, TNF‐α, and IL‐6, which are essential for carcinogenesis.^93^, ^94^, ^95^ It has been speculated that TLR4 was downregulated and TLR2 was upregulated in CRC patients, and low expression of TLR4 in the invasive front predicts poor prognosis and metastatic disease.^96^ A meta‐analysis study provided empirical evidence that TLR4 may play an important role in colorectal carcinogenesis and be a promising potential biomarker for the early diagnosis of CRC.^97^ TLR4 is overexpressed in human and murine inflammation‐associated colorectal neoplasia, whereas TLR4−/− mice were markedly protected from colon carcinogenesis.^98^ Furthermore, SNPs in TLR‐9 could also serve as biomarkers for decision making in the treatment of females with CRC.^99^

Similarly, microbiota and TLRs have an influence on CRC pathophysiological mechanisms. LPS‐TLR4 could cross‐regulate β‐catenin pathway and NF‐κB signal pathway which lead to variable colon cancer biological response.^100^ Other studies also speculated that LPS‐TLR4 might participate in CRC via PI3K/Akt pathway.^101^ Huang et al. claimed that bacterial endotoxin enhances CRC cell adhesion and invasion through activating TLR‐4/NF‐κB‐dependent urokinase plasminogen activator system.^102^ Besides, circulating cell‐free DNA was shown to promote cancer progression through stimulation of TLR9‐MyD88 signaling and IL‐8 secretion in CRC.^103^ Additionally, *F. nucleatum* was found to be among the most studied bacteria in the underlying carcinogenesis of CRC. *F. nucleatum* was shown to increase the expression of IL‐1β, IL‐6, and IL‐8 and impact the microenvironment of CRC through a possible miRNA‐mediated activation of TLR2/TLR4.^104^ *F. nucleatum* was also shown to induce intestinal tumorigenesis in ApcMin/+ mice via a TLR4/p‐PAK1/p‐β‐catenin S675 cascade.^105^ Yu et al. have indicated that *F. nucleatum* induced CRC chemoresistance via TLR4/Myd88 signaling pathway and suppression of TLR4 or Myd88 in the CRC xenograft mice and reduced both tumor weight and volume.^106^ Additionally, Yang et al. reported that presence of *F. nucleatum* increases proliferation of CRC and tumor development in mice by activating TLR4/NF‐κB signaling.^107^ Taken together, *F. nucleatum* is a potential risk factor for CRC and TLR4 could be a potential target for the prevention and therapy of *F. nucleatum*‐related CRC.

## 4 CONCLUSION

As important components in immune recognition, TLRs detect intestinal microbes and their metabolites, primarily focusing on modulating inflammation. Pathogenic bacteria can activate TLR signaling pathways, eliciting immune inflammatory responses, while TLRs can also recognize ligands produced not only by pathogenic microorganisms but also by symbiotic ones. Under normal steady‐state conditions, commensal bacteria are recognized by TLRs, providing protection against gut injury and associated mortality, thus ensuring a persistent immune response in normal animals. Furthermore, TLR signal transduction plays a pivotal role in either promoting or suppressing inflammation to maintain host balance. TLR2 and TLR4 are involved in inflammatory induction, while TLR2/IL‐10 and TLR9 mediate immunosuppression to uphold gut homeostasis. From these perspectives, TLRs and microbiota collectively contribute to the equilibrium between inflammation and homeostasis.

The moderated immune response, induced by the crosstalk between gut microbiota and TLRs, protects the host against pathogenic microbiota. However, an enhanced and continuous immune response might impair functional cells, leading to cellular injury and malfunction, such as autoimmune diseases. Therefore, the immune response functions as a double‐edged sword. In essence, the interaction between gut microbiota and TLRs plays a vital role in maintaining host homeostasis and contributes to the development of metabolic, inflammatory, and malignant diseases. With the identification of disease‐related pathogens and probiotics, TLRs and gut microbiota may evolve into a promising avenue for the treatment of related diseases.

## AUTHOR CONTRIBUTIONS

**Luping Chen**: Conceptualization; writing—original draft. **Linfang Zhang**: Conceptualization; writing—original draft. **Hua Hua**: Investigation. **Li Liu**: Investigation. **Yuejian Mao**: Resources. **Ruirui Wang**: Conceptualization; writing—review and editing.
